# CDR2L Antibodies: A New Player in Paraneoplastic Cerebellar Degeneration

**DOI:** 10.1371/journal.pone.0066002

**Published:** 2013-06-18

**Authors:** Tilo W. Eichler, Cecilie Totland, Mette Haugen, Tor H. Qvale, Kibret Mazengia, Anette Storstein, Bjørn I. Haukanes, Christian A. Vedeler

**Affiliations:** 1 Department of Clinical Medicine, University of Bergen, Bergen, Norway; 2 Department of Neurology, Haukeland University Hospital, Bergen, Norway; 3 Center for Medical Genetics and Molecular Medicine, Haukeland University Hospital, Bergen, Norway; Temple University, United States of America

## Abstract

**Objective:**

Yo antibodies are associated with paraneoplastic cerebellar degeneration (PCD). We have characterized Yo sera by measuring CDR2 and CDR2L antibodies and the localization of their antigens.

**Methods:**

Forty-two Yo sera from patients with paraneoplastic neurological syndromes (PNS), 179 sera from ovarian and 114 sera from breast cancer patients without PNS and 100 blood donors were screened for CDR2 and CDR2L antibodies by radioactive immune assay (RIA). Fluorescence microscopy was also used to determine the presence of CDR2 or CDR2L antibodies by staining of HeLa cells transfected with CDR2 or CDR2L fused to green fluorescent protein (GFP). Confocal microscopy was further used to localize the CDR2 and CDR2L proteins.

**Results:**

RIA showed that 36 of the 42 Yo positive sera contained CDR2 and CDR2L antibodies whereas 6 sera contained only CDR2 antibodies. Five of the ovarian cancer patients had CDR2L antibodies and 4 of the breast cancer patients had either CDR2 or CDR2L antibodies. Only patients with both antibodies had PCD. RIA and staining of transfected cells showed similar results. Yo antibodies were not present in the 100 blood donors. Confocal microscopy showed that CDR2 and CDR2L were localized to the cytoplasm, whereas CDR2L was also present on the cell membrane.

**Interpretation:**

Yo sera usually contain CDR2 and CDR2L antibodies and both antibodies are associated with PCD. Since only CDR2L is localized to the cell membrane it is likely that CDR2L antibodies may be of primary pathogenic importance for the development of PCD.

## Introduction

Patients with paraneoplastic cerebellar degeneration (PCD) often harbour Yo antibodies which cross-react with antigens in tumours (often ovarian or breast cancer) and Purkinje cells in the cerebellum [1]. Yo antibodies may also be associated with other paraneoplastic syndromes such as encephalomyelitis and can also be seen with other tumours such as prostate and colon cancer [2].

PCD is characterised by rapid development of pancerebellar symptoms and loss of Purkinje cells [3]. Purkinje cell death has been shown to occur in rat cerebellar slice cultures after uptake of Yo antibodies [4], however, the mechanisms involved in the associated Purkinje cell death in PCD are unknown.

Yo antibodies react with a 62 kDa protein (454 amino acids), the cerebellar degeneration-related protein 2 (CDR2; Reference sequence NP_001793.1) [1], [5]. CDR2 has been shown to act during mitosis in mammalian tumour cells through interactions with c-myc [5]. There are other members of the CDR family, including CDR2L (CDR2-Like, HUMPPA; Reference sequence: NP_055418.2). CDR2L, which is probably a CDR2 paralog, has approximately 50% sequence identity to CDR2. The canonical CDR2L transcript encodes a protein of 465 amino acids which, similar to CDR2, contains three potential coiled-coil regions. The functions of these proteins are so far not known.

Given the high sequence identity between CDR2 and CDR2L, we asked if Yo antibodies could cross-react with both antigens. The CDR2L specific antibody HPA022015 (www.proteinatlas.org) shows strong staining in Purkinje cells while the CDR2 antibodies HPA018151 and HPA023870 show moderate and weak staining, respectively. We therefore hypothesise that Yo antibodies could be directed against both CDR2 and CDR2L with CDR2L being the primary target on Purkinje cells. This was supported by the Genevestigator gene expression search engine (www.genevestigator.com), indicating low to medium CDR2 expression potential in the nervous system (reference probeset 209501_at (mean value cerebellum: 2114), and medium to high CDR2L mRNA levels (reference probeset 213230_at (mean value cerebellum: 8690), both based on the human genome 47 k array and 47 samples included.

## Materials and Methods

### Patients

#### Ethics statement

The part of the project involving patient sera is based on the bio-bank Paraneoplastic neurological diseases (#484) and approved by the Regional Committee for Medical and Health Research Ethics in Western-Norway, Diagnostic markers of cancer (188.05). The retrospective study of patient records was also approved by the Regional Committee for Medical and Health Research Ethics in Western-Norway and the clinical data were part of a larger retrospective study on clinical correlations with onconeural antibodies (Storstein et al. 2011). For both the bio-bank and the retrospective study, the regional ethics committee as well as the Ministry of Health and Care Services specifically waived the need to obtain consent (verbal and written), due to the large number of included subjects and high number of deceased subjects. All participants were adults.

First, we screened 42 Yo positive sera (with antibodies against CDR2) sent to the Neurology Research Laboratory, Haukeland University Hospital, Bergen, Norway for CDR2L antibodies using a transcription-translation and immunoprecipitation (IP) technique. Subsequently, we screened sera from patients with ovarian (n = 179) and breast (n = 114) cancer, as well as 100 blood donors for CDR2L antibodies using the same IP technique. These blood donor sera have previously been examined for CDR2 antibodies [6]. Hospital medical records were reviewed retrospectively in those patients in whom CDR2 or CDR2L antibodies were detected. The diagnosis of paraneoplastic neurological syndrome (PNS) was based upon symptom evolution, clinical findings and results of supplementary investigations according to recommended criteria [7].

### Transcription-translation and Immunoprecipitation (IP) Technique

Full-length CDR2 (www.origene.com, CDR2 sequence transferred from vector RG204900 with C-terminal green fluorescent protein (GFP) to vector PS100001 with C-terminal myc-DDK tag) or CDR2L (www.origene.com, CDR2L sequence in vector RC206909 with C-terminal myc-DDK tag) were cloned into expression vectors with a T7 promoter. For the IP experiments, two polyclonal rabbit antibodies from Eurogentec (www.eurogentech.com) which were produced against CDR2 (two synthetic peptides corresponding to amino acid 123–138 (EELKSSGQGRRSPGKC) and amino acid 429–443 (CDEQRTKYRSLSSHS) of the human CDR2 sequence) and CDR2L (two synthetic peptides corresponding to amino acid 282–297 (APEADDPQPGRGDDLG) and amino acid 392–407+C (CICRDSSWRDLRGGEEG) of the human CDR2L sequence) were used as reference sera. The antibodies were specific and did not cross react in the IP assay. CDR2 and CDR2L patient antibodies were then tested by the IP technique using the recombinant proteins as antigens [6], [8].

Antibody avidity was measured by adding urea to the antibody-antigen complex in the IP assay and high avidity was defined if 8 M urea did not destroy the antibody–antigen complex [9].

### Cell Cultures

HeLa cells (ATCC-CCL-2) were chosen as a cell-line with a human background that can easily be transfected with a high reproducibility of the results. Cells were grown in DMEM, supplemented with 10% FCS (Sigma), L-Glutamine (PAA, M11–004) and Pen Strep (Sigma, P4333). cDNA for full-length CDR2 (www.origene.com, CDR2 sequence transferred from vector RG204900 with C-terminal GFP into vector PS10019 with N-terminal GFP or vector PS100001 with C-terminal myc-DDK tag) or CDR2L (www.origene.com, CDR2L sequence transferred from vector RC206909 with C-terminal myc-DDK tag into vector PS10010 with C-terminal GFP) were used to transfect HeLa cells following the standard protocol for Lipofectamine2000 (Invitrogen, 11668–019). Transfected cells were fixed and immunocytochemically labelled 24 h after transfection.

### Immunocytochemistry (IC)

HeLa cells (ATCC-CCL-2) were grown on poly-L-Lysine coated glass-coverslips (10 mm). Immunocytochemical staining of HeLa cells with patient sera and antibodies against CDR2 and CDR2L was performed as described elsewhere [10]. All sera and antibodies were diluted in blocking solution, namely 0,2% gelatine in PBS. Three patient sera were chosen according to the findings from the IP: one positive for only CDR2, one positive for only CDR2L and one positive for both CDR2 and CDR2L, each used in a dilution 1∶200. Anti myc (Origene, TA50014) was used 1∶300, anti CDR2L (Biosite, ARP39113_P050) was used 1∶600. Secondary antibodies are coupled to Alexa Fluor (AF) 488 or 594 (Invitrogen, anti-rabbit-AF488 A11008, anti-rabbit-AF594 A11012, anti-human-AF594 A11014, anti- mouse-AF488 A11001) were used 1∶200. Coverslips were mounted with ProLong Gold with DAPI (Invitrogen, P36931) or MOWIOL without DAPI.

### Imaging

Imaging of HeLa cells was performed with a Zeiss Axiovert M200 fluorescence microscope with a 40x objective and TILL photonics monochromator or Leica TCS SP5 with Blue diode 405, Argon laser 488, Helium/Neon I laser 594, objective HCX PL Apo 63x.

### Immunohistochemistry and Immunoblot

Patient sera with CDR2 and/or CDR2L antibodies were tested by immunofluorescence using rat cerebellar tissue or immunoblot using recombinant onconeural proteins including CDR2 (Ravo Diagnostika, Freiburg, Germany, PNS003/48 [2]). Sections were also treated with high temperature citrate buffer to obtain antigen-retrieval.

Absorption experiments with recombinant CDR2 or CDR2L proteins obtained from the IP technique were performed as recently described [11].

Post mortem sections of cerebellum from a patient with no neurological disease or cancer were incubated with polyclonal antibodies against CDR2 (C-terminal) (Sigma, HPA023870), CDR2L (C-terminal) (Sigma, HPA022015) and CDR2L (N-terminal) (Biosite, ARP39113_P050). The immunoperoxidase staining followed the standard procedures at the Department of Pathology, Haukeland University Hospital.

### Western Blot

We subjected extracts of human cerebellum (150 µg) as well as lysates of recombinant CDR2 or CDR2L (from the IP test) to a 10% SDS-PAGE and performed Western blotting [11]. The membranes were first incubated with the polyclonal anti-CDR2 or polyclonal anti-CDR2L antibodies used in the immunohistochemical experiments and then with the anti-rabbit HRP antibody (DAKO, P0217). The reactions were developed by the ECL Plus western blotting detection system (RPN2132) (www.gelifesciences.com) and then read in a luminicent image analyzer.

## Results

### CDR2L and CDR2 Antibodies in Patient Sera

Among 42 sera previously tested for paraneoplastic antibodies, 36 were positive for both CDR2L and CDR2 antibodies using the IP technique (Table 1) and all 36 sera had high avidity for CDR2L and CDR2. Of these 36 sera, 3 were negative by line blot with recombinant CDR2 and 5 were negative by immunofluorescence staining of rat cerebellar tissue, also with sections treated with the antigen-retrieval protocol.

**Table 1:** Patients with both CDR2 and CDR2L antibodies.

			CDR2	CDR2L	P.cell	CDR2
No	Cancer	PNS	IP	IP	IF	LB
1	Colon	PCD +neuropathy	+	+	−	−
2	Ovary	PCD	+	+	+	+
3	Ovary	PCD	+	+	+	+
4	Ovary	PCD	+	+	+	+
5	Ovary	PCD	+	+	+	+
6	Ovary	PCD	+	+	+	+
7	Ovary	Myelopathy	+	+	+	+
8	Ovary	PCD	+	+	+	+
9	Ovary	PCD	+	+	+	+
10	Breast	PCD	+	+	+	+
11	Ovary	PCD	+	+	+	+
12	Ovary	PCD	+	+	+	+
13	Tube	PCD	+	+	+	+
14	Ovary	PCD	+	+	+	+
15	Tube	PCD	+	+	+	+
16	Unknown	PCD	+	+	+	+
17	Ovary	Unknown	+	+	+	+
18	Breast	PCD	+	+	+	+
19	Ovary	PCD	+	+	+	+
20	Tube	PCD	+	+	+	+
21	Ovary	PCD	+	+	+	+
22	Uterus	PCD	+	+	+	+
23	Ovary	PCD	+	+	+	+
24	Breast	PCD	+	+	+	+
25	Ovary	PCD	+	+	+	+
26	Uterus	PCD	+	+	+	+
27	Unknown	PCD	+	+	+	+
28	Unknown	PCD	+	+	+	+
29	Unknown	PCD +myelopathy	+	+	+	+
30	Ovary	No	+	+	−	+
31	Ovary	No	+	+	+	+
32	Breast	No	+	+	−	−
33	Unknown	Unknown	+	+	+	+
34	Unknown	Unknown	+	+	−	+
35	Unknown	Unknown	+	+	−	−
36	Unknown	Unknown	+	+	+	+

P. cell = Purkinje cell staining.

IP = Immunoprecipitation.

IF = Immunofluorescence.

LB = Line blot.

PNS = Paraneoplastic neurological syndrome.

PCD = paraneoplastic cerebellar degeneration.

Twenty eight of the 36 CDR2L and CDR2 antibody positive patients had PNS; 27 had PCD (two of them also had neuropathy or myelopathy) and one had isolated myelopathy. Three patients had no known PNS and no clinical information was available from five. Of the 36 CDR2L and CDR2 antibody positive patients, 18 had ovarian, 5 uterus (including 3 Fallopian tubes), 1 colon and 4 breast cancer; in 8 patients we had no information on a possible underlying tumour (Table 1).

We found 8 patients with only CDR2 antibodies, of which 6 were from the 42 routine sera. Two of the 8 patients had neuropathy, 5 had no known PNS and for one patient we had no clinical information. They all had different cancers; pharyngeal, prostate, uterus, colon, lung cancer or astrocytoma (Table 2). Two CDR2 sera were from the breast cancer materials with no PNS.

**Table 2:** Patients with either CDR2 or CDR2L antibodies.

			CDR2	CDR2L	P.cell	CDR2
No	Cancer	PNS	IP	IP	IF	LB
1	Pharynx	Neuropathy	+	−	−	−
2	Astrocytoma	No	+	−	−	−
3	Prostate	No	+	−	−	−
4	Uterus	Unknown	+	−	−	−
5	Lung	No	+	−	−	−
6	Breast *	No	+	−	−	−
7	Breast*	No	+	−	−	−
8	Colon	Neuropathy	+	−	−	−
9	Ovary *	No	−	+	−	−
10	Ovary *	No	−	+	−	−
11	Ovary *	No	−	+	−	−
12	Ovary *	No	−	+	−	−
13	Ovary *	No	−	+	−	−
14	Breast *	No	−	+	−	−
15	Breast *	No	−	+	−	−

* sera from the breast and ovarian cancer material.

P. cell = Purkinje cell staining.

IP = Immunoprecipitation.

IF = Immunofluorescence.

LB = Line blot.

PNS = Paraneoplastic neurological syndrome.

In 7 patients, we found only CDR2L antibodies; 5 of these were from the ovarian cancer and 2 from breast cancer cohort, and none had PNS. All of the CDR2 or CDR2L positive sera had low antibody avidity and none of them were positive by line blot or with immunofluorescence, including antigen retrieval protocol. None of the 100 blood donors were positive for CDR2L or CDR2 antibodies.

### Cell Cultures

**Figure 1:**
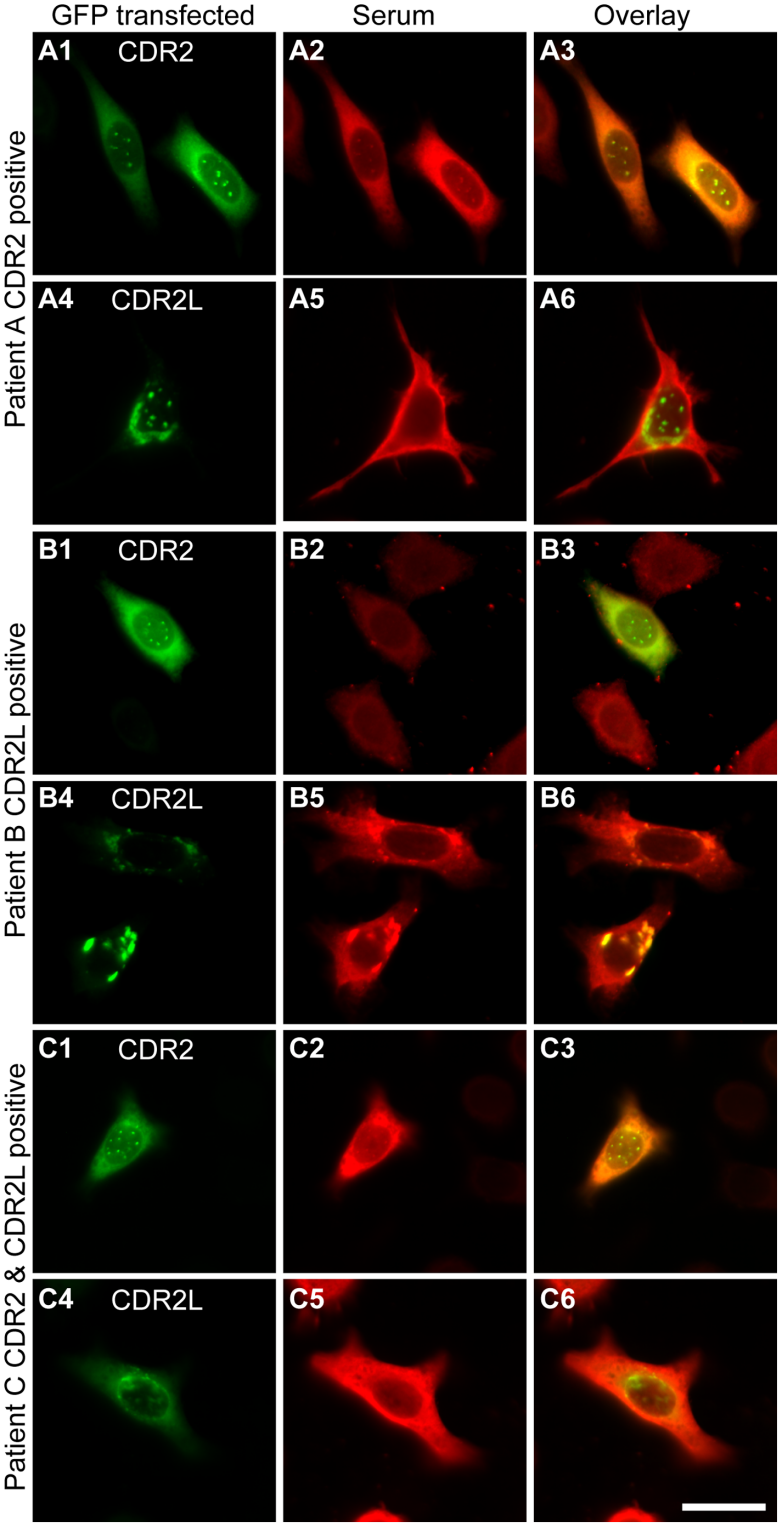
Three patient sera with different Yo antibody composition are shown. The first serum reacts with CDR2 (A1–3), but not CDR2L (A4–6). The second serum reacts with CDR2L (B4–6), but not CDR2 (B1–3). The third serum reacts with both CDR2 (C1–3) and CDR2L (C4–6). The first row shows HeLa cells transfected with CDR2 fused with GFP (A1, B1, C1) or CDR2L fused with GFP (A4, B4, C4) in green. The second row shows the immunocytochemical labelling of the transfected cells with patient serum and a secondary antibody goat-anti-human Alex Fluor 594 in red. The third row shows the overlay of GFP fluorescence and antibody-labelling, co-localization appears in yellow. Note that the first serum shows strong co-localization with CDR2 (A3), but not CDR2L (A6), the second serum shows strong co-localization with CDR2L (B6), but not CDR2 (B3), and the third serum shows strong co-localization with both CDR2 (C3) and CDR2L (C6). The results show that Yo sera can contain CDR2, CDR2L or both antibodies. Scale bar 25 µm.

Three patient sera that were shown to have different Yo antibody composition in IP experiments were chosen for IC labelling of HeLa cells expressing either CDR2-GFP (Fig. 1 A1, B1, C1) or CDR2L-GFP (Fig. 1 A4, B4, C4). All sera showed the same interaction in IP and IC. The CDR2 positive serum showed strong co-localization with CDR2 (Fig. 1 A1–3), but not with CDR2L (Fig. 1 A4–6). The CDR2L positive serum showed strong co-localization with CDR2L (Fig. 1 B4–6), but not CDR2 (Fig. 1 B1–3). The CDR2 and CDR2L double positive serum showed strong co-localization with both CDR2 (Fig. 1 C1–3) and CDR2L (Fig. 1 C4–6). The results support the IP results and show that Yo sera can contain CDR2, CDR2L or both antibodies.

**Figure 2:**
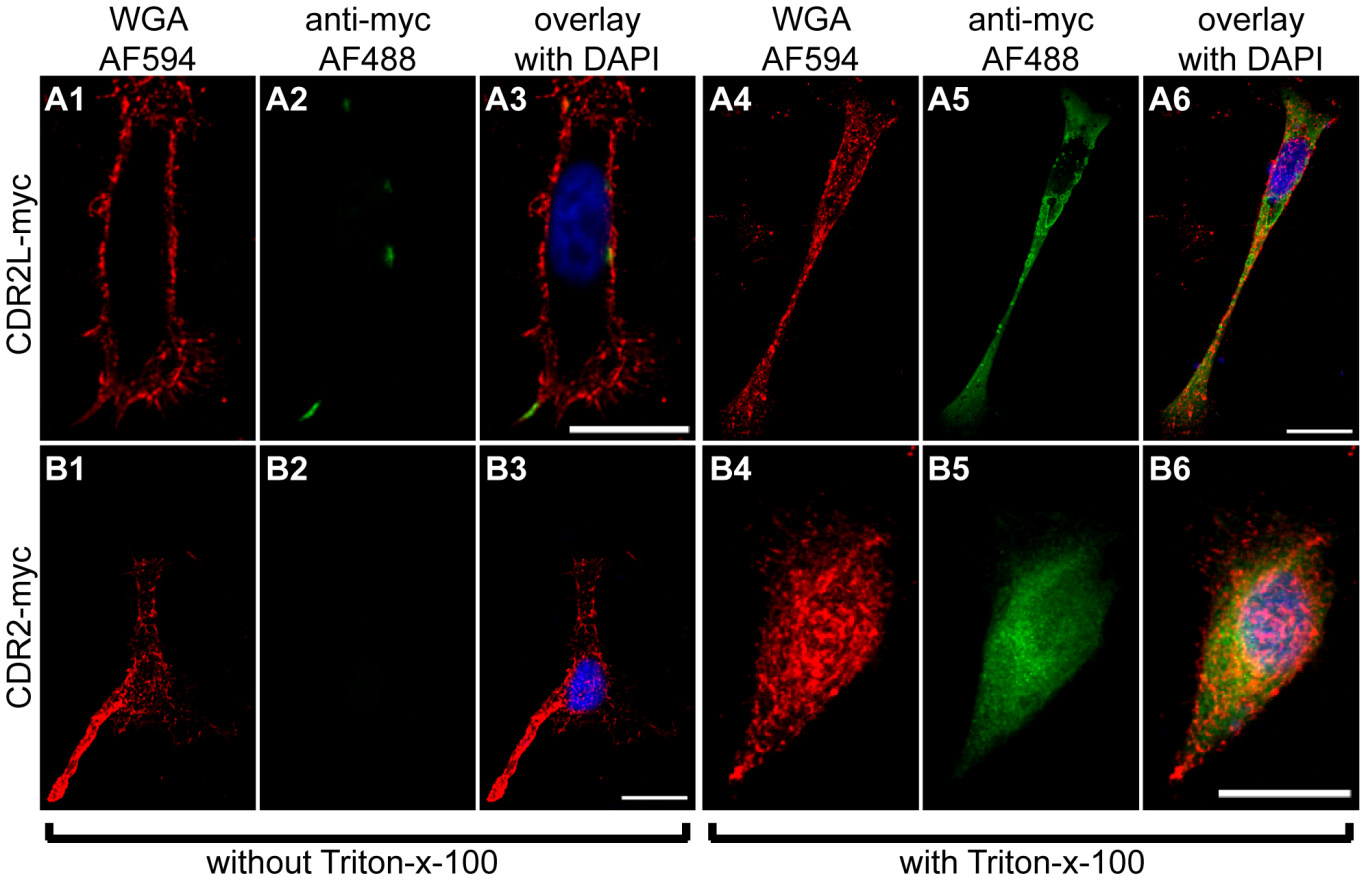
Localization of CDR2L and CDR2 in HeLa cells. HeLa cells transfected with CDR2L-myc (A1–6) or CDR2-myc (B1–6) were immunocytochemically stained with anti-myc antibodies (green, A2, A5, B2, B5). The membrane was visualized with wheat germ agglutinin (WGA, red, A1, A4, B1, B4). This was performed as a surface staining without addition of Triton-x-100 (A1–3, B1–3) or with Triton-x-100 to permeabilise the membrane (A4–6, B4–6). To show the membrane localisation of CDR2L, only selected slices of a z-stack were projected on top of each other, namely 16 (A1–3) or 23 (A4–6) slices. To show that CDR2 is not localised to the membrane, all slices covering the cells were superimposed, namely 55 (B1–3) and 40 (B4–6) slices. The overlays show that CDR2L can be detected in the membrane (A3), but not CDR2 (B3). Staining of non-permeabilised cells in B2 shows that CDR2 is not membrane-bound. However, the cells were successfully transfected with CDR2 as shown when the cells were permeabilised (B5). The results show that in the permeabilised cells, CDR2L (A5) is localized to the cytoplasm and cellular membrane, whereas CDR2 (B5) is only localised to the cytoplasm and nucleus. Scale bars 20 µm.

To determine if CDR antigens in intact cells are available to react with Yo antibodies, we performed a surface staining of HeLa cells transfected with CDR2-myc or CDR2L-myc (Fig. 2). Cells were fixed 24 hr after transfection and either treated with Triton-x-100 (Fig. 2 A4–6, B4–6) to permeabilise the plasma membrane or left untreated (Fig. 2 A1–3, B1–3). The plasma membrane was stained red with wheat germ agglutinin (WGA, Invitrogen) coupled to Alexa Fluor 594 (Fig. 2 A1, A4, B1, B4) and the CDR antigens detected with an anti-myc antibody and visualised with secondary antibody coupled to a green Alexa Fluor 488 (Fig. 2 A2, A5, B2, B5). In permeabilised cells, both CDR2-myc and CDR2L-myc could be detected inside the cells (Fig. 2 A6, B6). However, only CDR2L-myc could be detected on the cell surface (Fig. 2 A2), indicating that CDR2L is also membrane associated. To verify these results, cells transfected with CDR2L-myc were also surface-stained with anti-CDR2L antibody and anti-myc antibody (figure S1), resulting in a strong co-localisation of both antibodies, showing that the myc staining was specific for the transfected protein.

### Staining of Purkinje Cells

**Figure 3:**
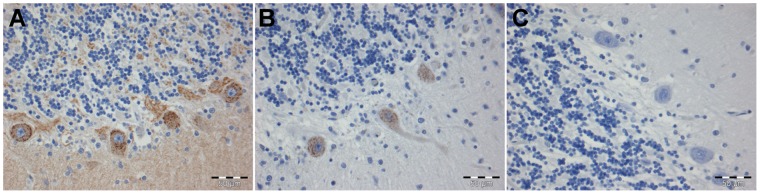
Staining of human Purkinje cells with CDR2 and CDR2L antibodies. Granular cytoplasmic reaction of human Purkinje cells with polyclonal antibody against the C-terminal (A) and the N-terminal (B) of the CDR2L protein. The C-terminal CDR2 antibody also gives some staining of the molecular and granular layers of the cerebellum (A). The polyclonal antibody against CDR2 did not stain the cerebellar sections (C). Scale bars 50 µm.

**Figure 4:**
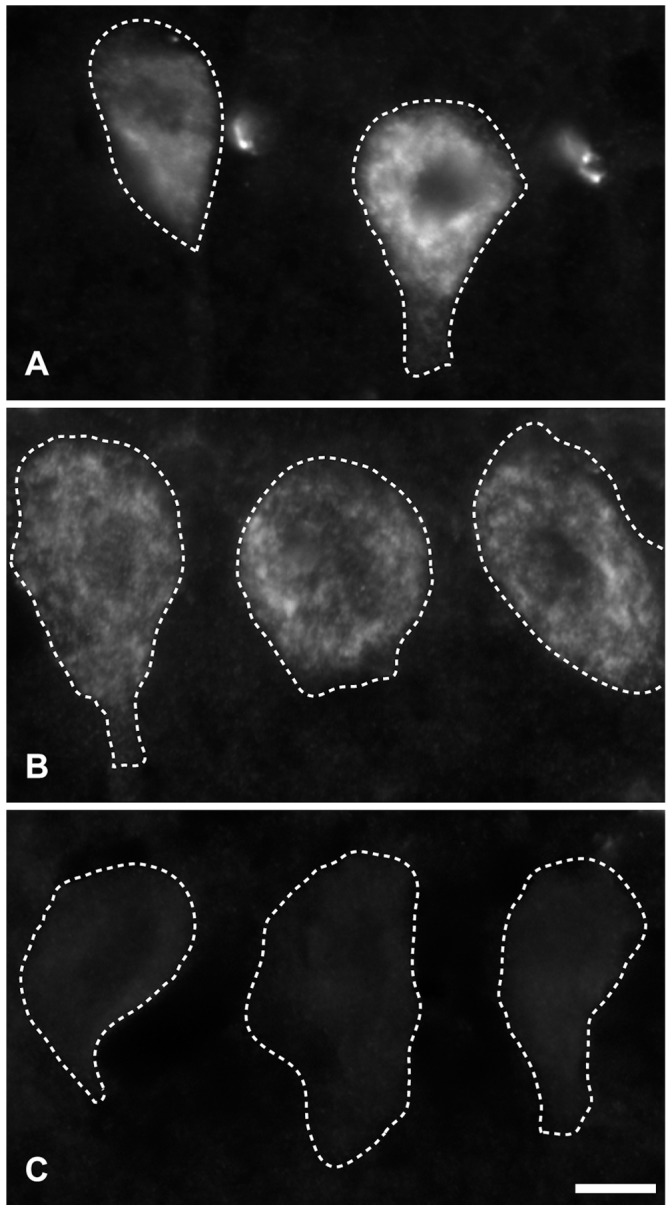
Yo positive serum containing CDR2L stains Purkinje cells. Serum from a patient with PCD and Yo antibodies (both anti-CDR2 and anti-CDR2L) shows granular cytoplasmic staining of Purkinje cells in rat cerebellar section (A). Similar staining of the serum was observed after absorption with recombinant CDR2 (B). However, no staining was observed when the serum was absorbed with recombinant CDR2L (C). Scale bar 10 µm.

Polyclonal CDR2L antibodies gave a granular cytoplasmic staining of human Purkinje cells, but no staining of these cells was seen by the CDR2 antibodies (Fig. 3). Neither of the antibodies stained rat cerebellar sections, even after antigen retrieval, while patient sera containing both CDR2 and CDR2L antibodies gave only cytoplasmic staining in rat Purkinje cells. Absorption experiments with recombinant CDR2 and CDR2L proteins showed that it was only the CDR2L antibodies in the patient sera that reacted with the Purkinje cells (Fig. 4).

### Western Blot

**Figure 5:**
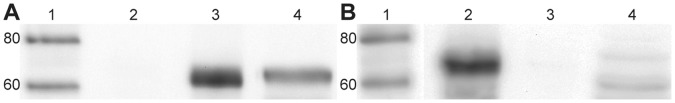
Test for specificity of polyclonal CDR2L and CDR2 antibodies on Western blot with recombinant antigens and human cerebellar extract. Western blot of human cerebellar extract incubated with polyclonal CDR2L antibody (A) or with polyclonal CDR2 antibody (B). In A, the CDR2L antibody does not react with recombinant CDR2 (A2), but with recombinant CDR2L (A3) and human cerebellum (A4). In B, the CDR2 antibody reacts with recombinant CDR2 (B2), but not with recombinant CDR2L (B3) and weakly with human cerebellum (B4). Lane 1 in both A and B is molecular weight standard.

The polyclonal CDR2 and CDR2L antibodies used for the Purkinje cell staining showed no cross reactivity between the recombinant CDR2 and CDR2L antigens (Fig. 5). The CDR2L antibodies gave a strong band of approximately 62 kDa in a Western blot of human cerebellum extract. There was also high reactivity with a protein of approximately 28 kDa with the antibody against the C-terminal of the CDR2L, but not with the antibody against the N-terminal of the CDR2L (data not shown). However, the CDR2 antibody showed only a weak band of approximately 62 kDa with the human cerebellar extract.

## Discussion

We found that 36 of 42 CDR2 positive patient sera also contained antibodies to CDR2L. The majority of the patients with both CDR2 and CDR2L antibodies had PCD, most often associated with ovarian cancer. The co-existence of these antibodies indicated that both antibodies had high avidity for their antigens. It is known that high avidity antibodies are more likely to be of pathogenic importance since they represent a more long-standing immune response [12].

When CDR2L or CDR2 antibodies occurred alone they had low avidity. Of the 15 sera that were positive only for one of these antibodies, only two of 8 CDR2 positive patients had possible PNS (neuropathy) and none with only CDR2L antibodies manifested a paraneoplastic syndrome. This suggests that the co-existence of these antibodies may be of relevance for the development of PNS, especially PCD. Low avidity CDR2 or CDR2L antibodies were all associated with cancer, but probably of most importance in the general tumour immune response and less for the development of PNS.

Although patient sera containing only CDR2 or CDR2L antibodies gave negative immunohistochemistry, these antibodies did stain HeLa cells transfected with their respective antigens. This is in line with the assumption that when occurring alone these antibodies have low avidity and mirrors findings with acetylcholine receptor (AChR) antibodies in which transfected cell assays are able to detect low avidity antibodies due to cross reactivity of the antigens on the cell surface [13].

We have shown previously that the IP test is more sensitive than both immunohistochemistry and line blot for the detection of paraneoplastic antibodies [2]. This may be due to antibodies in fluid-phase assays having better access to their epitopes [8]. In the present study, we found that 3 of the 36 CDR2 and CDR2L antibody positive sera were negative by line blot using recombinant CDR2, and 5 of the 36 sera were negative using immunohistochemical staining of rat cerebellar tissue.

HeLa cells transfected with CDR2L showed a different distribution of positivity than those transfected with CDR2. Localisation of CDR2L to the plasma membrane suggests a possible biological function similar to other membrane associated antibodies such as AChR and P/Q voltage-gated calcium channel antibodies (VGCC) that have been shown to be of pathogenic importance in myasthenia gravis and Lambert-Eaton myasthenic syndrome [14]. Whether CDR2L is also membrane linked in neurons is presently unknown. An electron microscopic study of a Yo positive serum from a patient with PCD showed an immune reaction localised to membrane-bound and free ribosomes in rat Purkinje cells [15]. Whether this patient serum contained both CDR2 and CDR2L antibodies was not investigated and so far, no similar studies have been performed using specific CDR2 or CDR2L antibodies.

The polyclonal CDR2L antibodies showed a granular cytoplasmic staining of the human Purkinje cells, similar to that described for Yo positive sera [1]. Despite this, we found that these CDR2L antibodies did not stain rat Purkinje cells, even after antigen retrieval. This is probably not due to differences between human and rat CDR2L, which show sequence similarity of approximately 93%, but more likely that the polyclonal antibodies contain low avidity antibodies for rat CDR2L. Similarly, patient sera containing only CDR2L antibodies did not stain rat Purkinje cells. In contrast, the combination of CDR2L and CDR2 antibodies increased their avidity and these patient sera stained rat Purkinje cells in a granular cytoplasmic manner in a similar way to the polyclonal CDR2L antibody staining of human Purkinje cells. Absorption experiments also showed that the Purkinje cell staining was due to the CDR2L antibodies.

The polyclonal CDR2 antibody did not react with human or rat Purkinje cells, even after antigen retrieval. Western blot studies of human cerebellar extract showed weak reactivity with the CDR2 antibody, but strong reactivity with the CDR2L antibodies. These results suggest that CDR2L is more highly expressed in human Purkinje cells than CDR2, findings that are in line with data showing that CDR2L mRNA levels are much higher than CDR2 levels in human cerebellum (biogps.org and www.genevestigator.com, probeset 213230_at (CDR2L) and 209501_at (CDR2).

According to the human protein atlas, both CDR2L and CDR2 are found in various human tumours (www.proteinatlas.org). We have shown previously that CDR2 is present in various tumours regardless of CDR2 antibody status [11]. In the present study, we also find that CDR2L is expressed in breast and ovarian cancer cells regardless of the presence of CDR2L antibodies in same patient sera (data not shown). CDR2L and CDR2 antibody production appears therefore to be dependent both on the tumour expression of these proteins, and probably also on regulation of the T and B cell responses in these patients [11].

Using sera from patients with PCD, Greenlee et al. (2010) showed that Yo antibodies can be taken up by cultured rat Purkinje cells. Our results suggest that it is likely that these sera contained both CDR2 and CDR2L antibodies, and that the co-expression of these antibodies is necessary for rat Purkinje cell staining. Moreover, based on our results, it is likely that the Yo antibody staining of the Purkinje cells in these experiments was due to CDR2L and not CDR2 antibodies. Furthermore, as we have shown in transfected cells, CDR2L is membrane-bound and probably therefore can mediate intracellular uptake of CDR2L antibodies.

The presence of high avidity CDR2L antibodies in patients with PCD and the presence of CDR2L in Purkinje cells make it tempting to speculate as to whether these antibodies are important in the Purkinje cell death that occurs in PCD [3]. The findings that Yo positive sera from patients with PCD, but not Yo negative sera, causes Purkinje cell death [4] point to this conclusion, but it remains to be seen if this is due to the CDR2L antibodies. Furthermore, cellular immune reactions may also occur in PCD. CDR2 specific cytotoxic T cells have been found in patients with PCD [16]. The expression of CDR2L in Purkinje cells makes it likely that such patients also harbour cytotoxic T cells against this antigen, and that a combined humoral and cellular immune reaction against CDR2L causes the degeneration of Purkinje cells in PCD.

## Supporting Information

Figure S1 **Surface staining of HeLa cells overexpressing CDR2L-myc.** HeLa cells transfected with CDR2L-myc (A1–3) were surface-stained with CDR2L antibody (red, A1) and myc antibody (green, A2). The surface staining was done without permeabilisation with Triton-x-100 (A1–3). Of the z-stack 15 slices were superimposed to show the localisation of CDR2L to the membrane. The overlay shows a strong co-localisation (yellow, A3) of both antibodies, indicating that the myc staining was specific for the transfected CDR2L protein. Scale bar 20 µm.
(TIF)
