# Supplementary figures and images for: CDR2L Antibodies: A New Player in Paraneoplastic Cerebellar Degeneration
Source: PLoS One. 2013 Jun 18;8(6):e66002. doi: 10.1371/journal.pone.0066002 (PMC3688866; doi:10.1371/journal.pone.0066002)

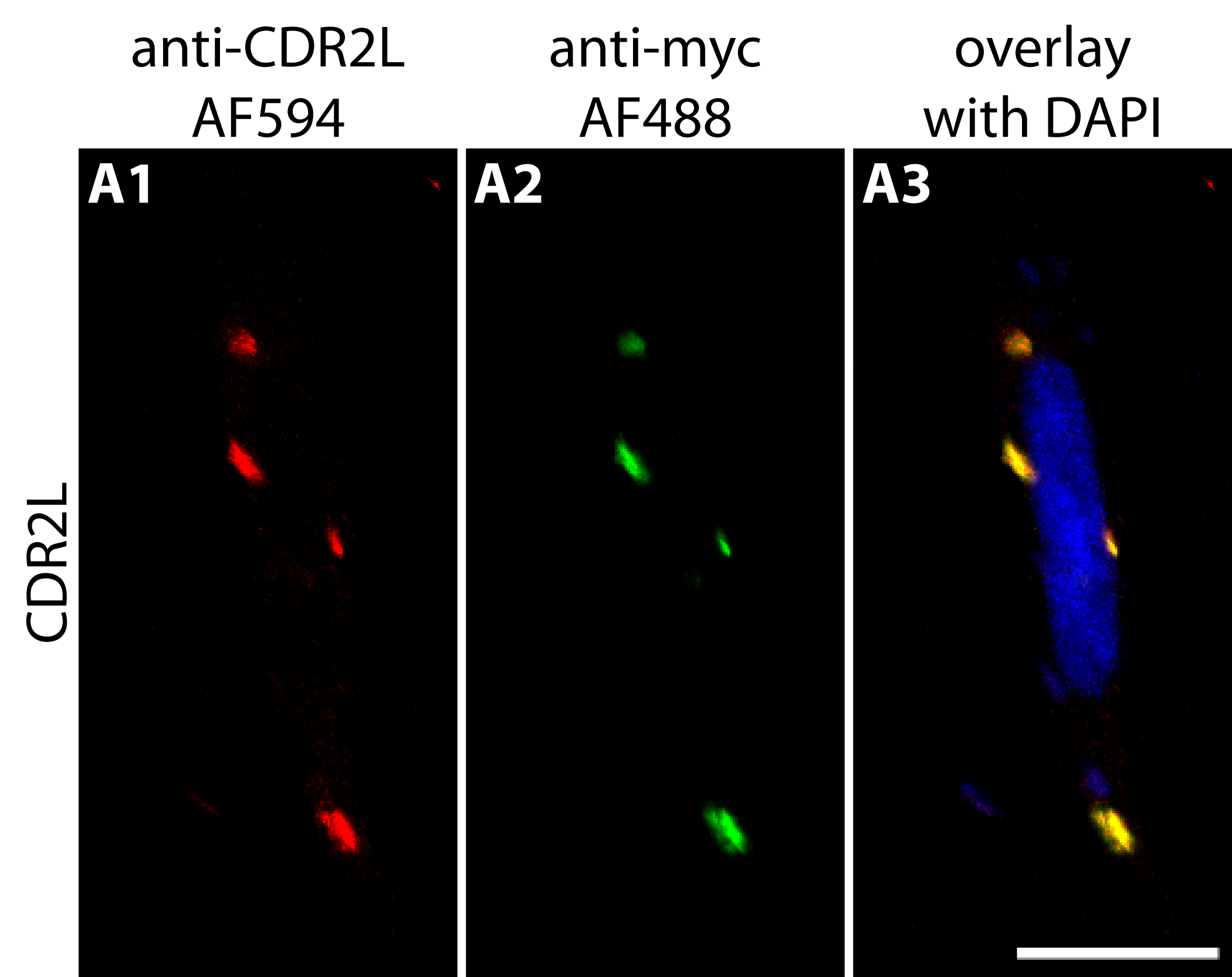

Supplement: Figure S1 — Surface staining of HeLa cells overexpressing CDR2L-myc. HeLa cells transfected with CDR2L-myc (A1–3) were surface-stained with CDR2L antibody (red, A1) and myc antibody (green, A2). The surface staining was done without permeabilisation with Triton-x-100 (A1–3). Of the z-stack 15 slices were superimposed to show the localisation of CDR2L to the membrane. The overlay shows a strong co-localisation (yellow, A3) of both antibodies, indicating that the myc staining was specific for the transfected CDR2L protein. Scale bar 20 µm. (TIF) [file pone.0066002.s001.tif]
